# Ursolic acid acetate and iso-mukaadial acetate bind to *Plasmodium falciparum* Hsp90, abrogating its chaperone function *in vitro*

**DOI:** 10.1007/s00210-024-02944-9

**Published:** 2024-01-22

**Authors:** Andani A. T Nndwammbi, Tendamudzimu Harmfree Dongola, Addmore Shonhai, Fortunate Mokoena, Ofentse J. Pooe, Mthokozisi B. C Simelane

**Affiliations:** 1 Department of Biochemistry, Faculty of Science, University of Johannesburg, Johannesburg, 2006 South Africa; 2 Department of Biochemistry & Microbiology, University of Venda, Thohoyandou, South Africa; 3 Department of Biochemistry, Faculty of Natural and Agricultural Science, North West University, Mmabatho, South Africa; 4 School of Life Sciences, University of KwaZulu-Natal, Durban, Westville 4000 South Africa

**Keywords:** *Plasmodium falciparum*, Iso-mukaadial acetate, Ursolic acid acetate, PfHsp90, Malaria

## Abstract

*Plasmodium falciparum* is the most lethal malaria parasite. Increasing incidences of drug resistance of *P. falciparum* have prompted the need for discovering new and effective antimalarial compounds with an alternative mode of action. Heat shock protein 90 (PfHsp90) facilitates protein folding and is a promising antimalarial drug target. We have previously reported that iso-mukaadial acetate (IMA) and ursolic acid acetate (UAA) exhibit antimalarial activity. We investigated the abilities of IMA and UAA to bind PfHsp90 by molecular docking and dynamics simulations. The *in silico* predictions were validated by biochemical assays conducted on recombinant PfHsp90. The interaction between the ligands and PfHsp90 was evaluated using ultraviolet-visible spectroscopy (UV-vis), Fourier transform infrared (FTIR), and surface plasmon resonance (SPR) analysis. The results obtained by docking calculations and MD dynamics simulation predicted that UAA and IMA preferentially bound to PfHsp90 via the N-terminal domain, with UAA binding more stable than IMA. UV-vis-based data suggest that PfHsp90 harbors buried aromatic amino acids, which were exposed in the presence of either IMA or UAA. In addition, data obtained using FTIR suggested that IMA and UAA destabilized the secondary structure of PfHsp90. Of the two compounds, UAA bound to PfHsp90 within the micromolar range based on surface plasmon resonance (SPR)-based binding assay. Furthermore, both compounds disrupted the holdase chaperone function of PfHsp90 as the chaperone failed to suppress heat-induced aggregation of the model proteins, malate dehydrogenase (MDH), luciferase, and citrate synthase *in vitro*. In addition, both compounds lowered the ATPase activity of PfHsp90. The molecular dynamics simulation analysis indicated that the docked complexes were mostly stable for 100 ns, validating the data obtained through the biochemical assays. Altogether, this study expands the repository of antiplasmodial compounds that have PfHsp90 among their possible targets.

**Supplementary Information:**

The online version contains supplementary material available at 10.1007/s00210-024-02944-9.

## Introduction

Malaria is an endemic life-threatening disease caused by female *Anopheles* mosquitoes carrying *Plasmodium* species. Of all *Plasmodium* species, *P. vivax*, *P. falciparum*, *P. malariae*, *P. knowlesi*, and *P. ovale*, *P. falciparum* is the most dangerous species that causes cerebral malaria, with 94% of cases reported in 2019 (2020). Children below the age of five and pregnant women are the most susceptible to the disease (2020). The continued discovery of drug-resistant parasites specifically towards artemisinin-based combination therapy (ACT) and chloroquine is particularly perturbing (Afonso et al. 2006), thus necessitating the need for novel antimalarial drugs. To date, most of the currently used antimalarial drugs have been developed from the plant’s secondary metabolites that have distinctive natural activity. *Warburgia salutaris* and *Mimusops caffra* are some of the plants found to contain antimalarial activity. From these plants, respectively, secondary metabolites known as iso-mukaadial acetate (IMA) and ursolic acid acetate (UAA) have been isolated, and they contain anti-*Plasmodium* activity (Simelane et al. 2013). In our hands, we have previously shown the antimalarial potential of drimane sesquiterpene isolated from *Warburgia salutaris* stem bark (2018).

Heat shock proteins (Hsps) are ubiquitous molecular chaperones mainly known for their roles in preventing and reversing protein misfolding. Several Hsps, including Hsp70 and Hsp90, are essential to the malaria parasite’s survival. Both Hsp70 and Hsp90 are considered possible antimalarial drug targets. Hsp90 and Hsp70 are highly conserved ATP-dependent molecular chaperones that fold proteins to ensure their maturation into functional conformations (Shonhai 2010). PfHsp70-1, a variant of Hsp70, is a cytosol-localized chaperone that cooperates with the cytosol localized isoform of Hsp90 of the parasite (Gitau et al. 2012). Both chaperones possess C-terminal EEVD motif that facilitates interaction with co-chaperones. PfHsp70-1 is thought to deliver specific proteins to PfHsp90 for further downstream folding (Gitau et al. 2012). PfHsp90 particularly folds proteins implicated in differentiation such as kinases and steroid hormone receptors (Shahinas et al. 2013).

PfHsp90 possesses approximately 69% identity to its human counterpart. As such, it is deemed not sufficiently selective as an antimalarial drug target. However, an inhibitor of PfHsp90, harmine, was demonstrated to potentiate chloroquine and artemisinin in a preclinical study where a significant reduction in parasitemia was observed in mouse models infected with *P. berghei* (Shahinas et al. 2012). Furthermore, targeting PfHsp90 seems an effective strategy due to its implication in *P. falciparum* drug resistance (Posfai et al. 2018). Highly conserved proteins are less prone to variation under selection pressure possibly, thus overcoming the hurdle of drug resistance (Shonhai 2010). In addition, despite the high level of sequence identity between the parasitic and human forms of Hps90, the two chaperones exhibit varied ATPase activities, suggesting the proteins have unique structure-function features (Pallavi et al. 2010; Posfai et al. 2018; Corbett and Berger 2010). Moreover, it was found that distinct expression of PfHsp90 can be correlated to different physiological states of malaria (Pallavi et al. 2010), confirming its role in regulating stage-specific development of the parasite. Additionally, previous studies have shown that known inhibitors of eukaryotic Hsp90 such as geldanamycin and its derivatives (17-AAG and 17-DMAG) PUH-71 bind PfHsp90 and inhibit parasite growth *in vitro* (Shahinas et al. 2013; Kim et al. 2004; Shonhai 2010; Pallavi et al. 2010).

Therefore, the identification of molecules that inhibit PfHsp90 may contribute new insights into the role played by PfHsp90 in the parasite transition from ring to trophozoite stage. Notably, both PfHsp70-1 and PfHsp90 are nucleotide regulated. However, it must be noted that PfHsp90 binds to ATP in dimer form. Furthermore, they share substrates. It is thus conceivable that both PfHsp70-1 and PfHsp90 may be targeted by nucleotide-inspired inhibitors targeting their nucleotide-binding motifs. Cockburn et al. (Cockburn et al. 2011) argued that compounds such as geldanamycin, which have structural similarities to purine analogs (ATP), may potentially inhibit PfHsp70-1 and PfHsp90 (Pallavi et al. 2010). Furthermore, polymyxin B, which was first demonstrated to inhibit Hsp90 (Gitau et al. 2012), was previously shown to inhibit PfHsp70-1 (Shahinas et al. 2012). To this end, in the current study, we explored the prospects of IMA and UAA (both known inhibitors of PfHsp70-1) as possible inhibitors of PfHsp90. To answer this question, we used *in silico* and biochemical approaches. Over the past few decades, *in silico* applications have become indispensable in molecular biology and biomedical fields. Computational methods such as molecular docking and modeling simulations have been used to validate and map out exact sequences involved in traction studies and accelerate drug discovery (Salomane et al. 2021; Schrödinger 2019; Bohórquez et al. 2003). The knowledge generated here may lead to a new set of inhibitors or scaffolds that can be used in the synthesis of inhibitors targeting PfHsp90 and inhibitors that can specifically inhibit the progression from *the P. falciparum* parasite ring to the trophozoite stage.

## Materials and methods

### In silico

#### Preparation of the protein

The three-dimensional coordinates of N-terminal PfHsp90 in complex with ADP (PDB code 3K60) (Corbett and Berger 2010), the only available 3D structure of PfHsp90, were retrieved from the protein databank: https://www.rcsb.org/ (Huang et al. 2015; Burley et al. 2019), preprocessed, and refined using protein preparation wizard in Schrödinger 2021-2 (Sastry et al. 2013; Schrödinger 2019). Erroneous atomic representations of the crystal structure were fixed by adding missing side chains, assigning correct bond orders, capping the protein termini, creating disulfide bonds, and removal of water molecules. Hydrogen bonds were optimized and tautomeric states at pH 7.4 were equilibrated using OPLS 2005 force field.

#### Preparations of ligands

The ligands: geldanamycin (GDA) and ursolic acid acetate (UAA), were downloaded from PubChem (https://pubchem.ncbi.nlm.nih.gov) in structure data file (sdf) format, and iso-mukaadial acetate was drawn using 2D sketcher in Maestro v12.8 (Schrödinger 2019). Each ligand (2D structure) was converted to its lowest energy 3D conformation using the LigPrep module of Maestro v12.8 in Schrödinger 2021-2 (Schrödinger 2019). In brief, the geometries of the ligands were optimized by assigning them appropriate protonation states (Shelley et al. 2007). The ligands were then optimized using OPLS4 force field, generating possible ionization states at a target pH of 7.4 +/− 2.0 using EPIK. Stereoisomers were computed to retain specified chiralities at most generating 32 per ligand Schrödinger (2021).

#### Induced fit docking (IFD)

Induced fit docking (IFD) was implemented to predict the affinity and binding modes UAA, IMA, and GDA to PfHsp90. IFD is a flexible docking protocol used on glide energy terms for initial scoring of a protein-ligand complex. Then, prime refinement module for accurate scoring (Schrödinger 2019; Zininga et al. 2017; 2006; 2021) and prime to refine them using an automated process from a Python script. The whole process is repeated several times producing a final list of poses ranked according to prime energy (2021) and glide XP empirical scoring functions, accurately predicting the ligand binding modes and the resulting structural changes of the receptor. To dock compounds UAA, IMA, and GDA to PfHsp90, a grid box of the binding site for the prepared structure of PfHsp90 was generated considering the co-crystallized ligand ADP as a centroid. ADP was removed and the residues Asn37, Arg98, and Phe124 were selected to define the side chains of residues located within a 5 Å distance from the ligand; the prime refinement step was employed. After the initial docking of each ligand, up to 20 poses were selected for further refinement using the extra precision (XP) mode (Sherman et al. 2006).

#### Molecular dynamics

The Desmond module of Schrödinger 2021-2 was used to conduct molecular dynamics simulation (MDS) analysis (2006) The purpose of the MDS was to assess the stability and estimate the dynamic behavior of each complex of the PfHsp90 and the three ligands (GA, IMA, and UAA). In detail, top scoring docked poses of PfHsp90 and ligands were individually prepared for MDS by placing them in a single-point charge (SPC) explicit orthorhombic box with a buffer distance of 10 Å. The system was solvated with transferable intermolecular potential 3P (TIP3P) water model and neutralized by addition of 0.15 M NaCl and additional Na+/Cl− ions. The particle-mesh Ewald method was used to calculate the long-range electrostatic interactions. Short-range van der Waals and Coulomb interactions were cut off at 9.0 Å radius. The minimization of the solvated system was performed utilizing OPLS-2005 forcefield (2021) parameters followed by relaxation. The Berendsen NVT ensemble was used to simulate the system by maintaining pressure (*P* = 1.01325 bar) and temperature (*T* = 300 K) using Nosè–Hoover chain thermostat and Martyna–Tobias–Klein barostat methods. Trajectories were recorded step by step every 50 ps. The NPT ensemble was initiated following the simulation process which runs for 100 ns production. MDS workflow was run performed remotely at the Centre for High Performance (CHPC, CSIR, and Cape Town).

### In vitro

The coding sequence of PfHsp90 plasmid was synthesized from Gene Scripts Biotech Corporation (Nanjing, China), and all other chemicals were purchased from Sigma-Aldrich (Pty) Ltd. (South Africa), Inqaba Biotechnical Industries (South Africa), and Thermo Fisher Scientific (Pty) Ltd. (South Africa) unless otherwise stated.

#### Expression of recombinant PfHsp90

The DNA segment encoding PfHsp90 (Plasmodb. Id; PF3D7_0708400) was cloned into pET28a(+) using Xho1 and Nco1 restriction enzymes. pET28a (+) _PfHsp90 plasmid was transformed in *E.coli* BL21 (DE3). Single colony containing the transformed plasmid was inoculated in 100-mL LB broth (1% w/v sodium chloride, 0.5% w/v yeast, and 1% w/v tryptone) enriched with 50 μg/mL kanamycin and grown at 37 °C for overnight shaking at 200 rpm. The overnight culture was sub-inoculated in 1-L fresh LB broth (1:10) containing 50-μg/mL kanamycin. The culture was grown at 37 °C until it reaches optical density at 600-nm wavelength (OD600) 0.4–0.6 at 140 rpm shaking incubator (Merck). The protein expression was induced by 0.5 mM IPTG for 24 h at 20 °C. Hourly samples were collected for 5 h post induction and harvested at 13,600 × *g* for 10 min at 4 °C. The pellet was resuspended in 1X Phosphate Buffer Saline (PBS) (137 mM NaCl, 2.7 mM KCl, 8 mM Na_2_HPO_4_, 1. 46 mM KH_2_PO_4_). The expression was analyzed by 12% sodium dodecyl sulfate-polyacrylamide gel electrophoresis (SDS-PAGE), stained with Coomassie brilliant blue, and visualized with ChemiDoc™ MP Imaging system (Bio-Rad, USA) and further analyzed by western blot using a monoclonal anti-Histidine (His6)-horseradish peroxide-conjugated antibody from mouse (Thermo Fisher Scientific Inc., SA).

#### Purification of recombinant PfHsp90

For purification, cells were harvested 24 h post induction at 13,600 × *g* at 4 °C, resuspended in phosphate lysis buffer (500 mM NaCl, 25 mM sodium phosphate pH 7.4, 20 mM imidazole) containing 1 mM PMSF, 30 μg/mL lysozyme, and 1 mM EDTA. The cell lysate was frozen at −80 °C overnight. The cells were retrieved and thawed on ice, and 0.1% (v/v) poly (ethyleneimine) (PEI) was added to precipitate nucleic acids followed by sonication for 30 s (3 cycles) at 60 Hz. The sonicated cell lysate was centrifuged for 20 min at 13,600 × *g* at 4 °C. The supernatant was incubated in nickel-charged nitrilotriacetic acid resin (Ni-NTA) (Thermo Scientific) beads for overnight at 4 °C. Flow through was collected, column was washed with wash buffer A (500 mM NaCl, 25 mM sodium phosphate pH 7.4, 25 mM imidazole) twice and once with wash buffer B (500 mM NaCl, 25 mM sodium phosphate pH 7.4, 80 mM imidazole), and protein was eluted with elution buffer A (500 mM NaCl, 25 mM sodium phosphate pH 7.4, 250 mM imidazole) twice and once with elution buffer B (500 mM NaCl, 25 mM sodium phosphate pH 7.4, 500 mM imidazole). The efficiency of recombinant protein purification was analyzed by 12% SDS-PAGE and western blot using a monoclonal anti-Histidine (His6)-horseradish peroxide-conjugated antibody from mouse (Thermo Fisher Scientific Inc., SA).

#### PfHsp90 secondary structure analysis using UV-vis spectroscopy

UV-vis spectroscopy was employed to analyze the secondary structure of PfHsp90, through aromatic amino acids (tyrosine, phenylalanine, and tryptophan) at 280-nm wavelength in the absence and presence of GA, IMA, and UAA. The UV-1800 spectrophotometer (Shimadzu, Japan) was used to monitor secondary confirmational change spectra at wavelength between 200 and 400 nm with control of UV-Probe software (version 2.7). Briefly, 2.5 μM PfHsp90 in the absence and presence of 5 mM and 10 mM of IMA and UAA respectively, was incubated for an hour at room temperature. 5-mM and 10-mM GA was used as positive control in place of IMA and UAA.

#### Fourier transform infrared spectroscopic analysis of PfHsp90

2.5-μM dialyzed PfHsp90 was incubated in the absence and presence of antimalarial compound IMA and UAA prepared in 100% dimethyl sulfoxide (DMSO). The reaction was performed in 5 mM IMA and UAA compound concentration and incubated for an hour at room temperature (≃ 25 °C) for protein-ligand complex formation. 5-mM GA was used for positive control. FT-IR measurements were done using IRAffinity-1S (Shimadzu, Japan) at room temperature. The IR spectral signals readings were done between 600 and 4000 cm^−1^ at resolution of 4 with 32 scans. The data was analyzed with Origin 8.5 software.

### ATPase activity assay

2.5 μM of recombinant PfHsp90 was incubated for 30 min with HDKM buffer (10 mM HEPES-KOH; pH 7.5, 0.5 mM dithiothreitol (DTT), 100 mm KCl, 2 mM MgCl_2_) room temperature (≃25 °C). Reaction was initiated by adding various ATP concentrations (0–2 mM) and incubated at 37 °C for 4 h. Fifty microliter of reaction solution was collected after each hour, and 50 μL of 10% SDS was added to stop the reaction. Fifty-microliter 1.25% ammonium molybdate and 50 μL of 9% ascorbic acid were added for color development. Inorganic phosphate release was monitored at 650 nm using a Synergy HT microplate reader (BioTek Instruments Inc., USA) controlled with Gen5 software (BioTek Instruments Inc., USA). Standard curve was extrapolated using di-sodium hydrogen phosphate (Na_2_HPO_4_) as a standard.

The assay was also assessed in the presence of 1–5 mM IMA and UAA with GA as positive control. The assay was monitored with the Synergy HT microplate reader (BioTek Instruments Inc., USA) controlled with Gen5 software (BioTek Instruments Inc., USA) at 650 nm. The ATPase activity of PfHsp90 was expressed as Pi released nmol/min/mg.

### Investigation of PfHsp90 chaperone activity

The chaperone activity of PfHsp90 was investigated by analyzing the ability of the recombinant protein PfHsp90 to suppress aggregation of malate dehydrogenase from porcine heart (Sigma-Aldrich (Pty) Ltd. (SA)) and luciferase from *Photinus pyralis* (Sigma-Aldrich (Pty) Ltd. (SA)). This was conducted using previously described by Salomane et al. (Salomane et al. 2021) with modifications. Briefly, 2-μM MDH and 1-μM PfHsp90 in assay buffer (50-mM Tris-HCl, pH 7.5, 100-mM NaCl) were mixed and incubated at 48 °C for an hour. 2-μM luciferase and 1-μM PfHsp90 in assay buffer (20-mM HEPES-KOH, pH 7.5, 50-mM NaCl) were incubated at 48 °C. 2-μM citrate synthase and 1-μM PfHsp90 in assay buffer (50-mM Tris-HCl, pH 7.5, 50-mM NaCl) were incubated at 48 °C. Protein aggregation was monitored for 60 min at 320 nm using the Synergy HT microplate reader (BioTek Instruments Inc., USA) controlled with Gen5 software (BioTek Instruments Inc., USA). The assay was also done furthermore to study the ability of iso-mukaadial acetate (IMA) and ursolic acid acetate (UAA) to inhibit the chaperone activity of PfHsp90 to suppress aggregation of MDH, luciferase, and citrate synthase. This was conducted using the described method above but with and without 5-mM IMA and UAA, respectively. 2-mg/mL BSA was used as a negative control instead of PfHsp90, and 5-mM GA was used as positive control instead of IMA and UAA.

### Surface plasmon resonance analysis

The possible interaction of PfHsp90 with both UAA and IMA was investigated using BioNavis Navi 420A ILVES multiparametric surface plasmon resonance (MP-SPR) system (BioNavis, Finland) as previously described (Muthelo et al. 2022). Briefly, PfHsp90 (ligand) was immobilized on a carboxymethyl dextran (3D CMD) chip through amine coupling. As analyte, IMA/UAA (analytes) were both injected at varying concentrations (0, 62.5, 125, 250, 1000, 2000 nM) over the 3D CMD chip at a flow rate of 50 µL/min. Filter-sterilized PBS was used as a running buffer. As a negative protein control, lysozyme was immobilized on 3D CMD chip in place of PfHsp90. To determine the steady-state equilibrium, association was allowed for 8 min and dissociation proceeded for 4 min, respectively, with temperature maintained at 25 ℃. Data Viewer (BioNavis, Finland) was used to resolve the sensorgrams. Signals generated in the channel representing buffer control were subtracted as background. Trace Drawer software version 1.8 (Ridgeview Instruments, Sweden) was used to estimate the binding affinities.

### Statistical analysis

The experiment was repeated three times from different batches of purified protein, and the mean and standard error of mean were calculated. GraphPad Prism 8.4.3 software was used to determine the statistical analysis of mean values among differential groups. The data was observed objected to one-way and two-way analysis variance (ANOVA). The *p* ≤ 0.05 was regarded statistically significant.

## Results

### Docking calculation indicates that UAA binds to PfHsp90 with greater affinity than IMA

**Fig. 1:**
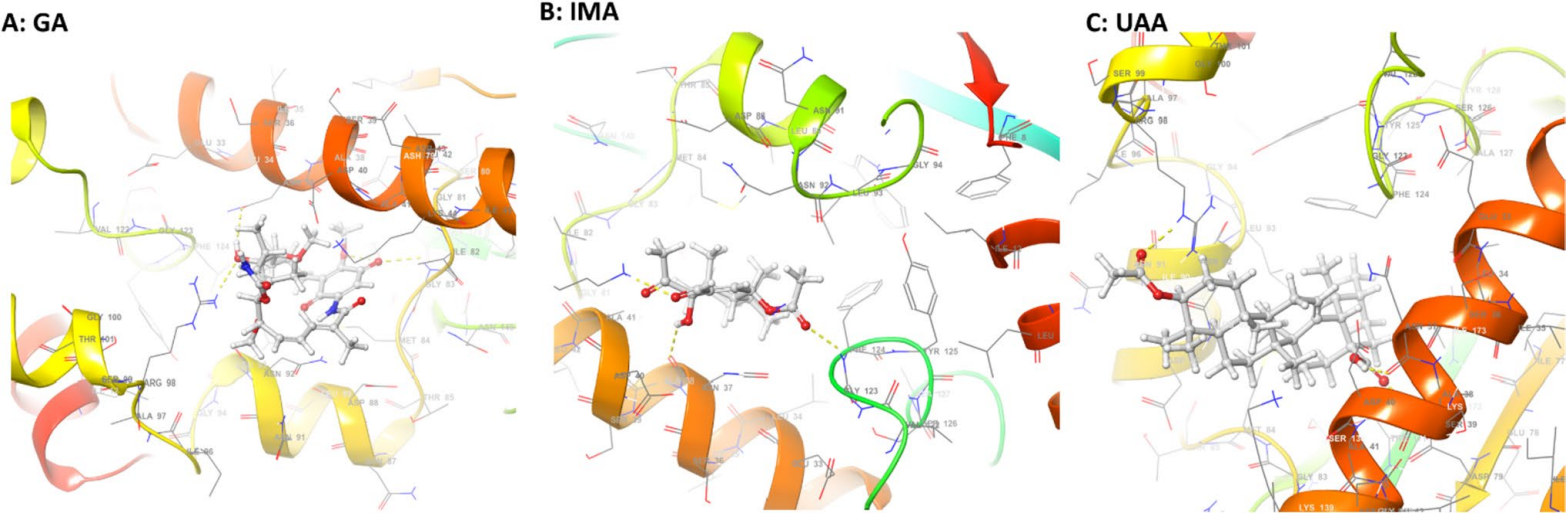
**A**–**C** Respective docking top scoring poses of GA, IMA, and UAA into the PfHsp90, N-terminal domain pocket and ligand interaction amino acids

IFD indicated that both IMA and UAA had acceptable binding energies −5.399 and −8.564 kcal/mol in comparison to a proven inhibitor of PfHsp90, GA, exhibiting a docking score of −7.170 kcal/mol (Table 1). Visual inspection of the interactions revealed that the three compounds displayed different modes of interactions with PfHsp90 (Fig. 6). The binding of GA into the PfHsp90 pocket is facilitated by four hydrogen bond contacts with GLY83, ASN37, ARG98, and THR171. Notably, GA makes significant hydrophobic interaction contact with residues LEU34, ALA38, ILE82, MET84, LEU93, and ILE173 within the 4 Å cut-off. IMA on the other hand formed three hydrogen bonds with ASN37, LYS44, and PHE124. UAA makes hydrogen bond contacts with ASN37, ASP79 and ARG98 and hydrophobic interactions with LEU34, ALA38, ALA41, MET84, PHE124, VAL136, and ILE 173 (Fig. 1).

**Table 1:** Docking scores and PfHsp90 residues predicted to make direct contact with ursolic acid acetate and iso-mukaadial acetate

Compound	Docking score (kcal/mol)	Glide E model (kcal/mol)	IFD score (kcal/mol)
GA	−7.170	−78.275	−516.53
IMA	−5.399	−53.732	−510.57
UAA	−8.564	−72.025	−511.62

**Fig. 2:**
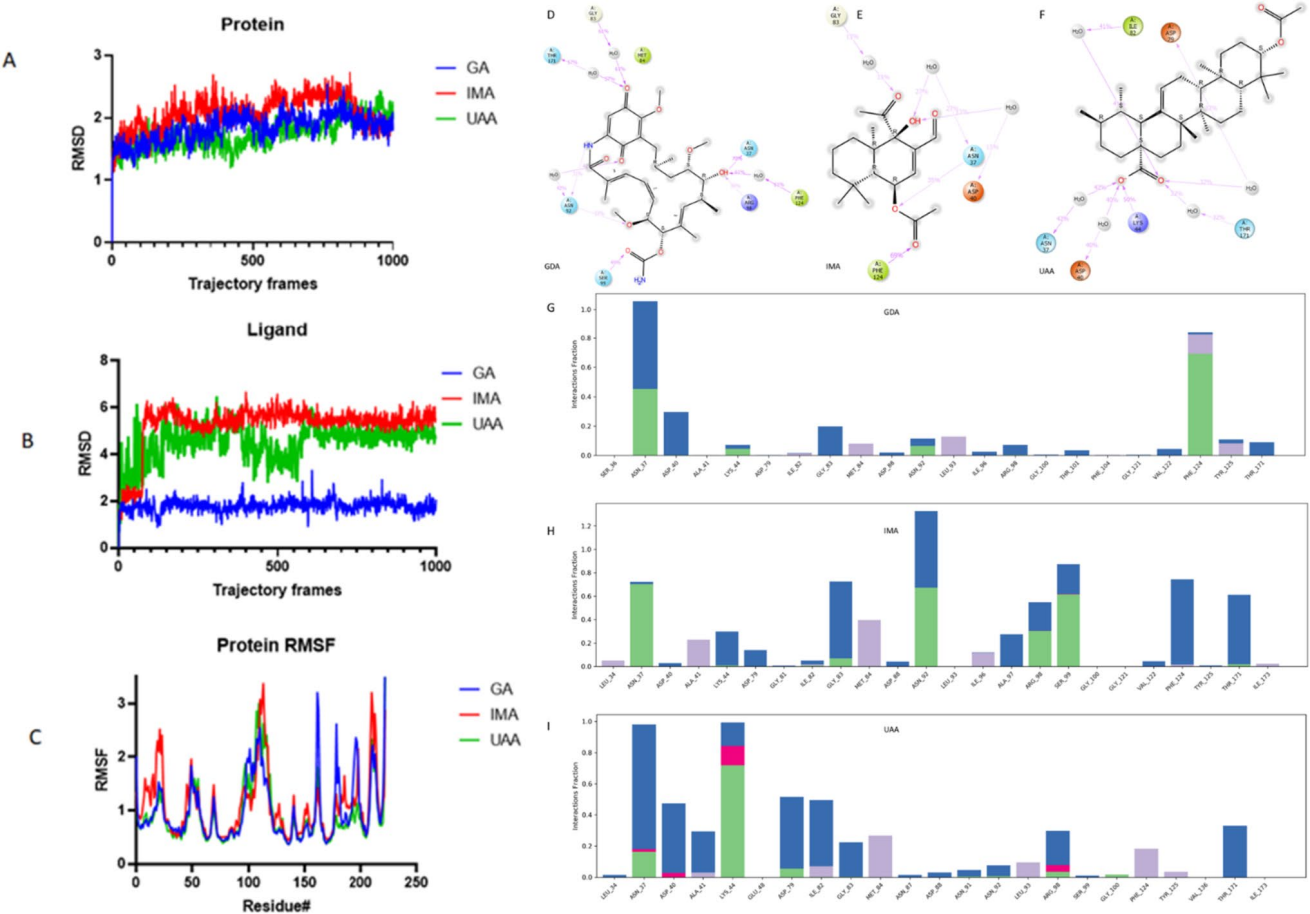
**A**, **B** At 100 ns, C-α atoms of the protein and ligand root-mean-square deviation (RMSD), respectively. Change in protein/ligand RMSD depicted by the left *Y*-axis over snapshot of trajectories. Change in ligand RMSD depicted by the right *Y*-axis over time. **C** Root-mean-square fluctuation (RMSF) of individual residues of the protein. Representation of **D**–**F** PfHsp90 and 2D ligand interactions and **G**–**I** histograms of the percentage interactions over the 100-ns simulation window

The Desmond package simulation analysis was used for post-processing of induced fit docking poses. MD trajectories captured at 0–100 ns were used to calculate the root-mean-square deviation (RMSD), root-mean-square fluctuation (RMSF), and protein-ligand interactions of the top scoring poses of GA, IMA, and UAA (Fig. 2).

The root-mean-square deviation (RMSD) indicates global changes in the receptor and ligand upon binding. There are fewer variations in the receptor PfHsp90 for all ligands, with RMSD values ranging 1.8–2.4Å (Fig. 2). Lower RMSD variations indicate the stability of the ligand in the binding pocket of the receptor, as can be seen for GA, which equilibrates at 38-ns RMSD of 2.0Å (Fig. 2A). Although considerably stabilized in PfHsp90 binding pocket, UAA displays a marginally higher RMSD 4.2 Å (Fig. 2B). It is possible that IMA is not well accommodated in the binding pocket of PfHsp90 based on the notably high RMSD of 6 Å (Fig. 2). Figure 2C displays local changes in the PfHsp90, less fluctuations on most of the protein except the loop segments (residue 110–145).

Ligand interaction diagram was critical binding mode of GA, IMA, and UAA analysis (Fig. 2D–I). It was revealed that study compounds formed hydrophobic contacts, hydrogen bond, ionic interaction, and salt bridges over the 100-ns MDS. Protein-ligand contact histogram was used to study interactions made for more than 30% of the simulation; a 0.1 value indicates 10% interactions fractions while values greater than 0.3 are considered as stable as complex molecule. The three study compounds formed hydrogen bonds and salt bridge contacts with ASN37 which is critical for ATPase activity of PfHsp90 (Corbett and Berger 2010). GA also made hydrogen bond contact with ASN92 and SER99 for 60%, 25%, and 55% (Fig. 2D, G), respectively. Furthermore, GA made water bridge with GLY83, PHE124, and THR171 for 70%, 78%, and 60% of the simulation time, respectively. The two phytochemicals exhibited different binding modes with IMA showing hydrogen bond and hydrophobic interactions with PHE124 (Fig. 2E, H). Meanwhile, UAA mainly interacts with ASN73 and THR171 via water-mediated hydrogen bonds (Fig. 2F, I).

### SPR analysis suggests that UAA binds to PfHsp90 with notable affinity

**Fig. 3:**
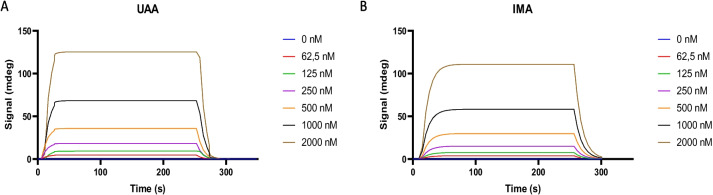
Both UAA and IMA exhibit variable affinities for PfHsp90

SPR data suggests that PfHsp90 bound to both UAA and IMA in a concentration-dependent fashion (Fig. 3). Of the two compounds, UAA demonstrated notable affinity for PfHsp90 (within the micromolar range) (Table 2). On the other hand, IMA bound to PfHsp90 with much less activity (10-fold lower affinity than UAA). The relative affinities exhibited by the two compounds are in sync with data obtained using in silico analysis (Fig. 1, Table 1).

**Table 2:** Binding affinities of the compounds obtained using SPR

Compound	Ka	Kd	KD	Chi^2^	*U* value
IMA	4.55*E*+03±36*e*2	8.81*E*−02±1.17*e*−5	1.94*E*−05±3.68*e*−6	7.9	6.2
UAA	1.18*E*+04±4.66*e*2	9.70*E*−02±7.93*e*−6	8.16*E*−06±3.24*e*−7	5.3	8.8

The SPR-generated sensorgrams show dose-dependent interaction of UAA/IMA with PfHsp90.

### PfHsp90 secondary structure analysis by UV-vis spectroscopy

**Fig. 4:**
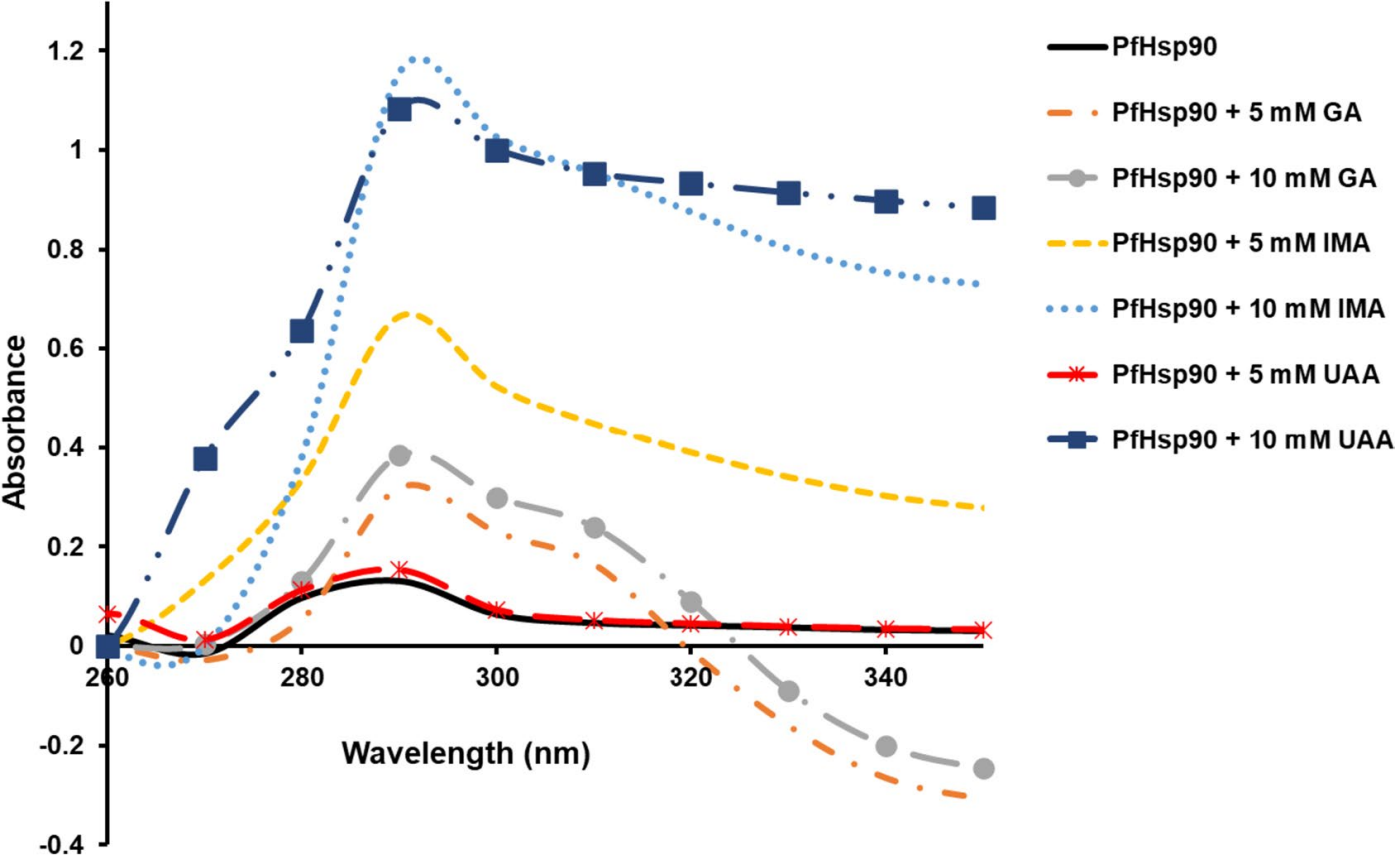
Secondary structure conformation analysis using UV-vis spectroscopy

The secondary structure of PfHsp90 was analyzed using UV-vis spectroscopy in the absence and presence of two concentrations of compounds (GA, IMA, and UAA). This was done by monitoring changes in aromatic amino acids, tryptophan, phenylalanine, and tyrosine under UV light. Absorbance increases between 270 nm and 300 nm showing the excitation of aromatic amino acid ring portion (Fig. 4). At the presence of compounds (GA, IMA, and UAA) at two concentrations (5 mM and 10 mM), PfHsp90 have more absorbance than PfHsp90 only with maximum excitation at 290 nm; this was due to an increase in the number of exposed aromatic rings (Fig. 4).

UV-vis spectrum analysis of PfHsp90 was also monitored in the absence and presence of GA, IMA, and UAA using two concentrations (5 mM and 10 mM). Spectra were monitored between 260 nm and 350 nm.

### Secondary structure analysis of PfHsp90 by Fourier transform infrared (FTIR)

**Fig. 5:**
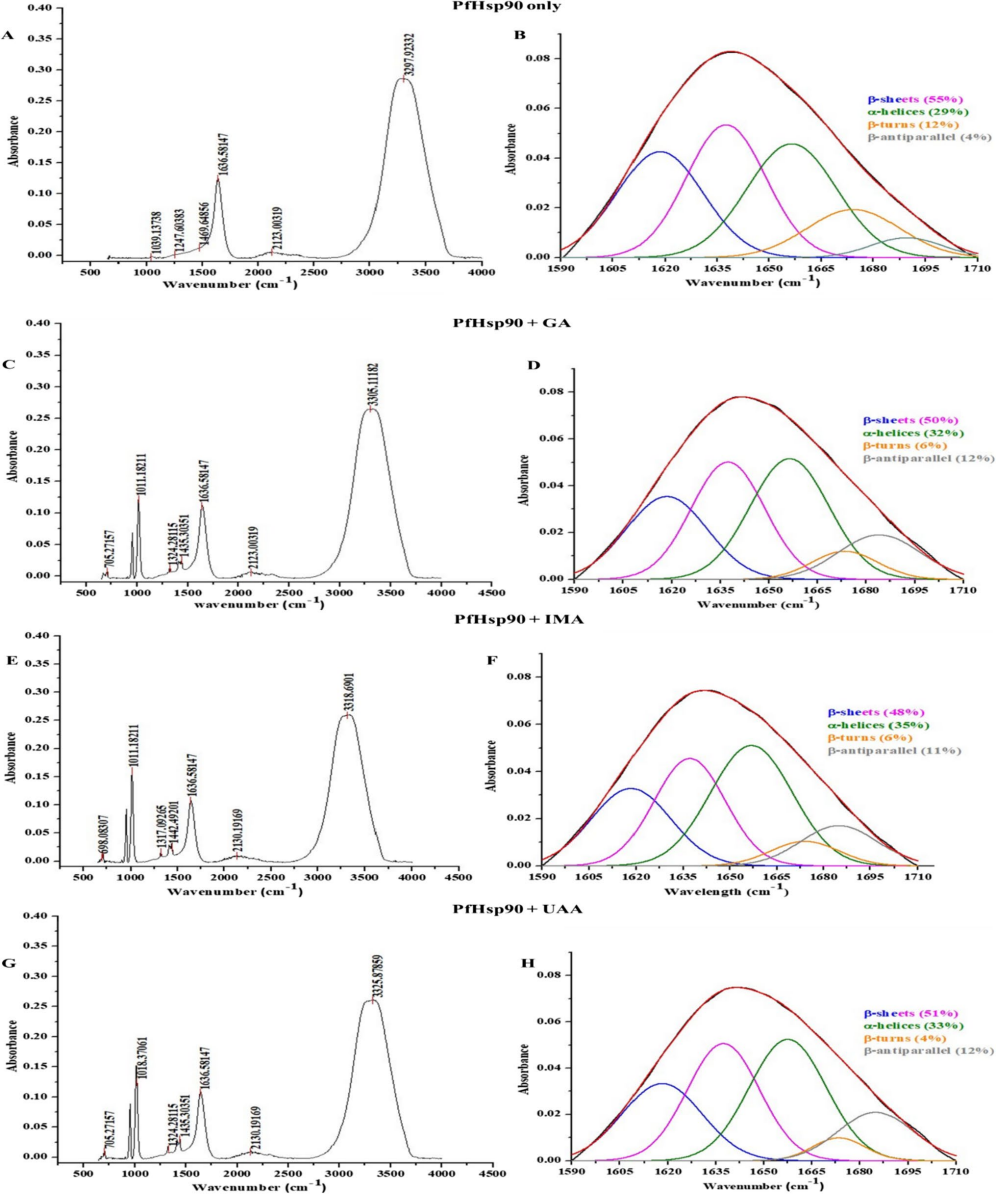
Representative FTIR spectra of PfHsp90. Spectrum analysis of amide groups from 600 to 4000 cm^−1^ (**A**, **C**, **E**, **G**) and deconvoluted and curve fitted amide I for different secondary structure elements between 1700 and 1600 cm^−1^ region (**B**, **D**, **F**, **H**)

Fourier transform infrared spectroscopy (FTIR) was employed to monitor shifting of spectra signals and dimension variation of amide groups, amide A, I, II, and III of PfHsp90 at the absence and presence of 5-mM GA, IMA, and UAA, respectively (Fig. 5). The monitoring of amide groups aimed to investigate the secondary structures, α-helix, β-sheet, β-turn, and β-antiparallel change by deconvolution of amide I band (Fig. 5).

The binding of any of the compounds (GA, IMA, or UAA) to PfHsp90 did not appear to result in band shifting with reference to amide I. However, band shifting was observed for amide A, II, and III. Amide A band shifts from 3297 cm^−1^ for free PfHsp90 to 3305, 3318, and 3325 cm^−1^ in PfHsp90-GA, PfHsp90-IMA, and PfHsp90-UUA complexes, respectively (Fig. 5C, E, G). The shifting was due to N-H stretch groups in PfHsp90. Amide II spectral shifting was observed from 1469 cm^−1^ in PfHsp90 only to 1435, 1442, and 1435 cm^−1^ in PfHsp90-GA, PfHsp90-IMA, and PfHsp90-UUA complexes, respectively. Shifting spectra of amide III were also observed from 1247 cm^−1^ in PfHsp90 only to 1324 cm^−1^ in PfHsp90-GA complex, 1317 cm^−1^ in PfHsp90-IMA complex, and 1324 cm^−1^ in PfHsp90-UUA complex.

Only in amide I band deconvolution, PfHsp90 was found to consist of α-helix (1656 cm^−1^: 29%), β-sheet (1618, 1637 cm^−1^: 51%), β-turn (1674 cm^−1^: 12%), and β-antiparallel (1684 cm^−1^: 4%) (Fig. 5). However, when PfHsp90 binds to interact with GA, which is a positive control, there was reduction of β-sheets from 51 to 50% and β-turn from 12 to 6% with a shift from 1674 to 1673. An increase in α-helicity was observed from 29 to 32% with β-antiparallel content increasing from 4 to 12%, concomitantly associated with a shift from 1688 to 1684 cm^−1^ (Fig. 5D). Upon the introduction of IMA or UAA, there was a significant increment of the α-helicity of PfHsp90 only (29%) to 48% and 51% from PfHsp90-IMA and PfHsp90-UAA, respectively. Notably, there was also reduction of β-turn content from 12 to 6% for PfHsp90-IMA and from 12 to 4% for PfHsp90-UAA complexes, respectively. By contrast, a significant increase was observed in α-helicity and β-antiparallel content. α-Helix increased from 29% in PfHsp90 to 35% in PfHsp90-IMA and 33% in PfHsp90-UAA complex, respectively. On the other hand, β-antiparallel composition increased from 4% in apo-PfHsp90 to 11% in PfHsp90-IMA complex and 12% in PfHsp90-UAA complex, respectively (Fig. 5F, H).

### The basal ATPase activity analysis of PfHsp90

**Fig. 6:**
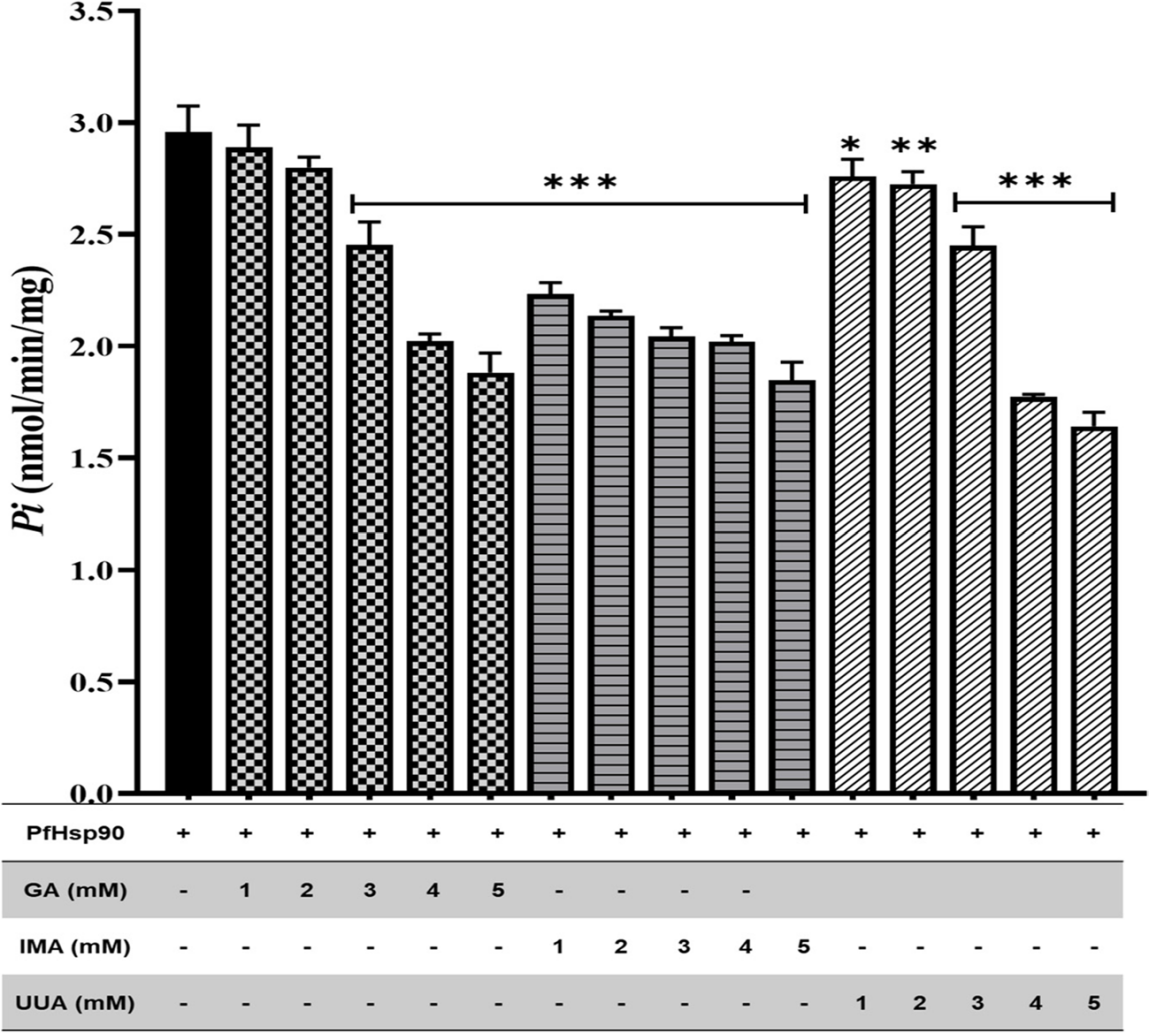
Dose-dependent basal ATPase activity analysis. Relative basal activity of PfHsp90 protein in the function of different GA, IMA, and UAA concentrations (1–5 mM) was monitored at 650 nm. The statistical significance *p*<0.0001 of each complex relative to PfHsp90 was done using a two-way ANOVA test

The effects of IMA and UAA the ATPase activity PfHsp90 were investigated. 1–5-mM GA was used as a positive control. The concentrations of compounds were varied from 1 to 5 mM, and the increase in compound concentration decreased ATPase activity rate (Fig. 6). The change of rate of ATPase suggests that these compounds interfere with PfHsp90 ATPase activity meaning the lesser Pi the more ATPase activity inhibition by compounds thus suggesting that IMA and UAA may bind to PfHsp90 N-terminal ATPase domain.

### UAA and IMA inhibit the chaperone activity of PfHsp90 in vitro

**Fig. 7:**
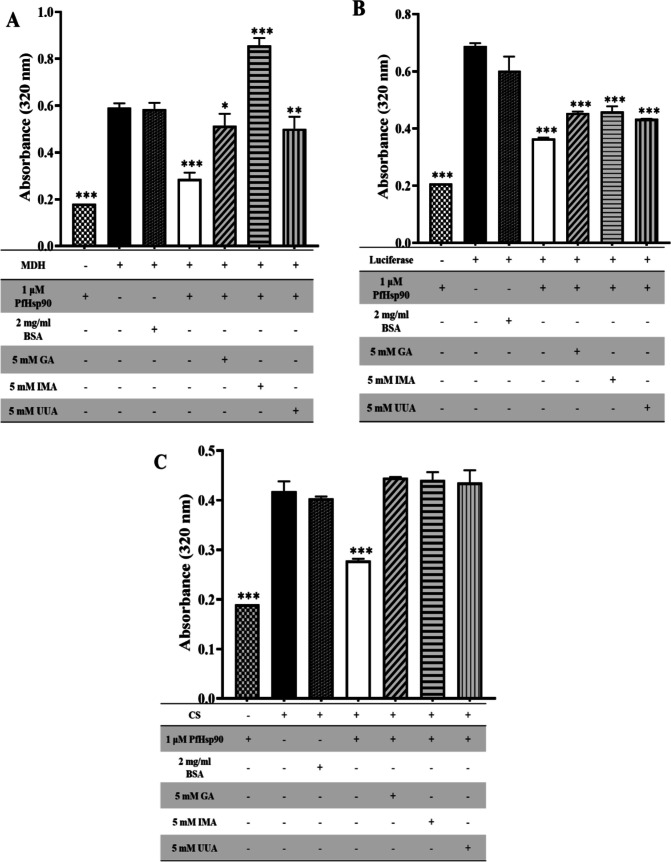
IMA and UAA inhibit PfHsp90 suppression chaperone activity. Heat-induced aggregation was monitored at 48 °C in vitro and measured at 320 nm. The ability of PfHsp90 to suppress aggregation of **A** MDH, **B** luciferase, and **C** CS was monitored in the absence and presence of 5-mM IMA and UAA, respectively. GA was used as positive control and 2-mg/mL BSA as a negative control. Statistical analysis of client proteins to others using one-way ANOVA test was significant with *p*<0.0001***

The ability of PfHsp90 chaperone activity to suppress client substrates, malate dehydrogenase (MDH), luciferase, and citrate synthase (CS) heat-induced aggregation was monitored at 320-nm reading absorbance. The results show that 1-μM PfHsp90 effectively suppressed the aggregation of all three proteins (Fig. 7A–C). The assay was repeated to determine the ability of study compounds, IMA and UAA, to inhibit chaperone activity of PfHsp90 from suppressing aggregation of all client proteins (MDH, luciferase, and CS), respectively (Fig. 7A–C). As expected, as a non-chaperone control, BSA did not suppress the aggregation of MDH. On the other hand, all three compounds, GA, IMA, and UAA, inhibited PfHsp90 chaperone activity (Fig. 7A–C). However, IMA increases the aggregation of MDH more drastically than GA and UAA (Fig. 7A, p<0.0001***).

## Discussion

Multidrug *P. falciparum* resistance has become a major problem. However, studies have identified compounds iso-mukaadial acetate (IMA) and ursolic acid acetate (UAA) that possess antiplasmodial activity (Simelane et al. 2013; 2018). Since Hsps are implicated in the development of parasite resistance to drugs, it is of great interest to target them for drug discovery. PfHsp90 is an ATP-dependent protein upregulated during the development of the parasite and interacts with PfHsp70-1 to fold client proteins to their native state in the presence of co-chaperone, PfHop (Pallavi et al. 2010; Zininga et al. 2015). In addition, IMA and UAA are known to bind PfHsp70-1 (Shonhai 2021), a functional partner of PfHsp90 (Gitau et al. 2012), inhibiting its chaperone function (Salomane et al. 2021). Therefore, the current study investigated PfHsp90 structural and functional activity in the presence of IMA and UAA.

PfHsp90 secondary structure was analyzed by UV-vis spectroscopy by monitoring aromatic amino acids, phenylalanine (Phe), tyrosine (Tyr), and tryptophan (Trp). These amino acids have significant quadrupole moments and can interact with cations (Bohórquez et al. 2003; Ruan and Rodgers 2004). At spectral region 245–270 nm, 265–285 nm, and 265–295 nm, Phe, Try, and Trp residue absorbance arises, respectively (Kueltzo et al. 2003). Generally, soluble folded protein has non-polar amino acids buried in the innermost far from the solvent-exposed surface (Lucas et al. 2006). However, Phe due to its non-polarity, the benzene ring is buried in the interior. At the same time, Try and Trp side chain phenol and indole, respectively, are differentially disseminated to the surface from the interior (Lucas et al. 2006).

In PfHsp90 only, UV-vis spectra exhibited an increase in absorbance between 270 and 300 nm, showing exposed Try and Trp aromatic amino acid rings with the highest peak at 290 nm (Fig. 5). Effects of IMA and UAA on PfHsp90 secondary structure aromatic amino acids were also studied. There was an increase in absorbance in protein-compound complexes as compound concentration increases compared to protein only at 270–300 nm, as observed with GA (Fig. 5). This suggests that GA, IMA, and UAA interact with PfHsp90 to expose the buried aromatic amino acid side chains; a similar effect was observed in the study conducted by Banumathy (Banumathy et al. 2003). In addition, a higher peak at 290 nm reflects increased exposure of Trp. FTIR was also used to explore the secondary structure of PfHsp90 either in the absence or presence of IMA/UAA. The reduction in the peak signal of PfHsp90 observed suggests the conversion of β-sheets to α-helix while β-turns were converted to β-antiparallel conformation. The data suggest that GA, IMA, and UAA do not interact with amide I of PfHsp90 since no spectral shift is observed which leads to the observed modulation of the secondary structure of the protein.

Furthermore, the basal ATPase activity of PfHsp90 and the ability of IMA and UAA to compete with ATP were investigated using a colorimetric assay. This was investigated by measuring inorganic phosphate released when ATP binds to PfHsp90. Protein: compound dose-dependent at constant ATP was performed, and results showed that an increase in compound (IMA and UAA) concentration decreased ATPase activity rate (Fig. 6). Hsp90 itself has a low ATPase activity. Understanding PfHsp90-specific inhibitor affinity is important for antimalarial drug development (Chua et al. 2012). From this study, it was observed that PfHsp90 exhibits low ATPase activity and low binding affinity. And we established that UAA displayed a higher affinity for PfHsp90 than IMA (Fig. 3). UAA works similarly to GA (Wang et al. 2016). Altogether, the disruption of PfHsp90 ATPase activity suggests that these compounds IMA and UAA bind to the N-terminal domain as GA is known to bind and inhibit Hsp90 at the N-terminal domain; it is worth noting that this could also be due to allosteric function. Moreover, our findings established that both IMA and UAA impair the ATPase activity of PfHsp90. These findings are in line with those of Salomane et al. (Salomane et al. 2021) which demonstrated that IMA binds to the N-terminal ATPase domain of PfHsp70-1 to inhibit its chaperone function.

The ability of PfHsp90 to suppress heat-induced aggregation of model proteins (MDH and citrate synthase) was investigated in vitro. From the results obtained, PfHsp90 was found to suppress the aggregation of client proteins MDH, luciferase, and citrate synthase (Fig. 7). IMA, UAA, and GA were all shown to inhibit PfHsp90 chaperone activity (Fig. 7). These data suggest that although GA binds to PfHsp90, it was incapable of suppressing the holdase chaperone activity of PfHsp90. These findings concur with those of Tavella et al. (Tavella et al. 2021).

The observed interaction of PfHsp90 with the compounds studied here was validated using wet lab experiments. We demonstrated that IMA and UAA both strongly docked onto the PfHsp90 receptor site (Fig. 1, Table 1). Several hydrogen bonds are associated with the formation of the PfHsp90-ligand complexes. Notably, hydrogen bonding via ASN37 was a common feature in all the complexes. This is in line with previous observations suggesting that ASN37 facilitates the ATPase activity of PfHsp90 (Kawaguchi et al. 2015; 2015). The dynamic behaviors of the complexes were evaluated by molecular dynamics run at 100 ns. RMSD provide valuable information regarding the dynamic deviation for ligand–protein complex when compared to their reference structure, and high RMSD values can be correlated to significant instability due to conformational changes (2012; Opoku et al. 2019; Erukainure et al. 2021). IMA and UAA showed notable RMSD values which were close to those reported for the inhibitor, GA (Wang et al. 2016). The data, therefore, suggest that the ligands were adequately accommodated with the studied receptor during the MD simulation time frames (Fig. 2).

## Conclusions

Malaria remains a major killer disease with resistance to current malaria therapies being reported worldwide (2020). As such, it is important to identify alternative antimalarial compounds and their targets. In the current study, we established that both IMA and UAA inhibit PfHsp90 chaperone function. However, UAA exhibited higher affinity for PfHsp90 than IMA. In this regard, our findings expand the library of antiplasmodial compounds targeting PfHsp90. In light of the essential role of this protein in the development of the parasite, the current knowledge contributes to the efforts to expand the target inhibitor database towards finding alternative antimalaria interventions.

### Supplementary Information

Below is the link to the electronic supplementary material.

Supplementary file1 (DOCX 646 KB)

## Data Availability

The data related to this article can be publicly available after the article accepted. The authors confirm that the samples of the compounds are available from the authors.
