# Supplementary material for: Ursolic acid acetate and iso-mukaadial acetate bind to Plasmodium falciparum Hsp90, abrogating its chaperone function in vitro
Source: Naunyn Schmiedebergs Arch Pharmacol. 2024 Jan 22;397(7):5179–92. doi: 10.1007/s00210-024-02944-9 (PMC11166764; doi:10.1007/s00210-024-02944-9)
Supplement: Supplementary file 1 — Supplementary file1 (DOCX 646 KB) [file 210_2024_2944_MOESM1_ESM.docx]

**Supplementary data**

**Recombinant PfHsp90 successful expression and purification**

Recombinant PfHsp90 protein was successfully expressed in *E. coli* BL21 (DE3) cells (Figure 9A). The PfHsp90 protein production was analysed by 12% SDS-PAGE and western blot using an anti-polyhistidine (α-His) antibody. SDS-PAGE analysis showed an increase in PfHsp90 expression level as time increases at approximately 100 kDa (Figure 4A, top panel). It was then confirmed by western blot, which showed PfHsp90 expressing at 100 kDa with a possible breakdown below 95 kDa due to *E. coli* system proteolysis (Figure 9A, bottom panel). Western blot also confirmed leaky expression prior to induction. It was found that PfHsp90 is soluble (Figure 9B, lane S), therefore nickel affinity chromatography was employed for native purification of the recombinant protein. Some PfHsp90 protein was lost in flow-through and washes (Figure 4B, lane FT and W1). PfHsp90 was successfully purified at approximately 0.25 mg/ml (Figure 9B). Western blot was used further to identify PfHsp90 by using monoclonal anti-Histidine (His6)-horseradish peroxide conjugated antibody from mouse (Figure 9B, bottom panel).

**
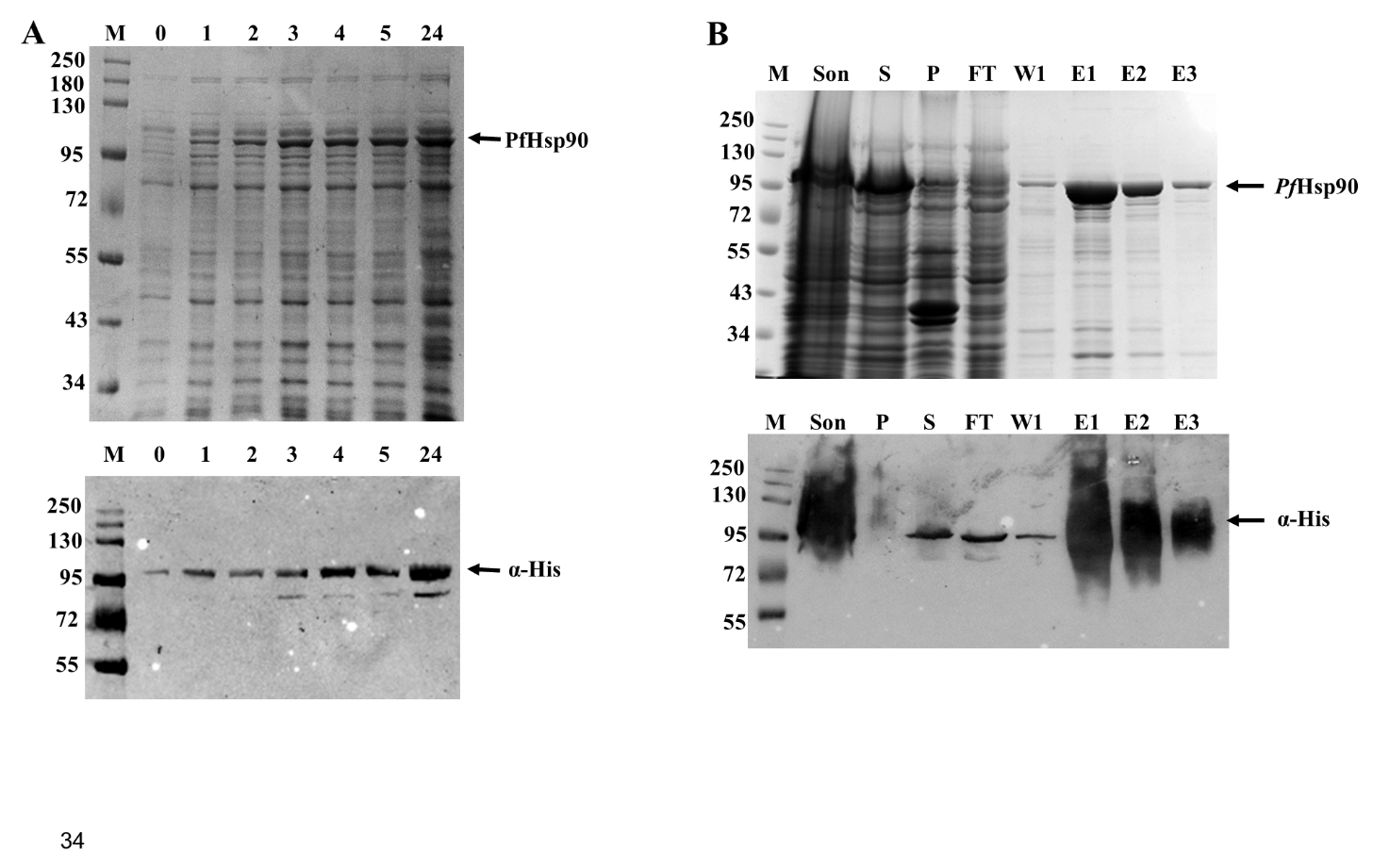
**

**Figure 9**. Expression and purification of recombinant PfHsp90

(A) Recombinant PfHsp90 protein was expressed in *E. coli* BL21 (DE3), (B) purified using nickel affinity chromatography and analyzed by 12% SDS-PAGE (Top panel) and western blot using anti-His antibody (bottom panel). Lane M-protein molecular marker (kDa), lane 0- whole cell lysate from cells transformed with pET28a (+) _PfHsp90 before induction with IPTG. Lane 1-5: samples taken after every hour post-induction, while lane 24 represents the sample collected after 24 hours post-induction. Son-sonicated lysate, S-supernatant fraction, P-pellet fraction, FT-flow through, W1-wash 1, E1-E3 – elution 1, elution 2, and elution 3, respectively.
